# Perceived effort for motor control and decision-making

**DOI:** 10.1371/journal.pbio.2002885

**Published:** 2017-08-18

**Authors:** Ignasi Cos

**Affiliations:** Universitat Pompeu Fabra, Theoretical and Computational Neuroscience Group, Center for Brain and Cognition, Barcelona, Catalonia, Spain

## Abstract

How effort is internally quantified and how it influences both movement generation and decisions between potential movements are 2 difficult questions to answer. Physical costs are known to influence motor control and decision-making, yet we lack a general, principled characterization of how the perception of effort operates across tasks and conditions. Morel and colleagues introduce an insightful approach to that end, assessing effort indifference points and presenting a quadratic law between perceived effort and force production.

## How much effort is effortful?

Because of the redundancy of the motor system, movements aimed at a target may vary in different ways, with adjustments to duration, curvature, or path length made as a function of the task condition. When there is a defined target to reach for, farther targets require movements of longer duration and by extension, presumably, of greater effort [1]. However, experimental data has also shown that when the target is ambiguous [2–4], movement duration remains relatively constant regardless of distance, therefore implying an increase of movement intensity to preserve temporal duration. But do these strategies obey a principle bound to minimize subjective effort?

The perception of effort is a major constraint for movement generation and also for deciding between potential movements [5,6], and its abnormal perception is a relevant hallmark for a broad set of disorders such as depression, chronic fatigue, Parkinson’s, or Huntington’s disease [7]. However, the main hurdle in grasping the specifics of effort is that it cannot be directly assessed. We can objectively measure the metabolic energy consumption associated with performing a movement as well as its relationship with specific choices of movement parameters and with decisions between movements. Furthermore, metabolic energy, movement proficiency, and subjectively perceived effort are known to be interrelated, as studies of motor learning often report that movements become energetically more efficient and are perceived as less tiring as athletic proficiency increases [8]. However, despite all the evidence, we still lack a general formalism capable of describing how effort is globally perceived and how it relates to behavior across tasks and conditions [9].

Research by Morel and colleagues published in *PLOS Biology* [10] has shed light on this matter by introducing a novel paradigm to assess perceived effort by probing participants’ choices between resistive movements of different costs. They asked human participants to compare 2 different actions and to choose the least effortful one, while moving the handle of a robotic manipulator that resisted the participants’ applied force. The authors dissected space and time by establishing 2 main results: first, duration, not distance, increases the perception of effort on resistive reaching movements; second, the relationship between the force exerted and its associated perceived effort follows a quadratic law.

The first result is consistent with previous evidence showing that energy-related costs are influential in deciding between movements [11], sometimes even when these costs are detrimental to task performance [12]. In addition, their results also suggest that the previously recorded influence of distance on decisions between movements is likely to be caused by the adjustments to movement duration rather than by distance per se [8]. Whether these results generalize to other movements remains to be established, as their results are constrained to resistive reaching movements.

## Optimal movement: From energy to utility

A first hypothesis that relates movement parameters and decisions to energy postulates the optimization of metabolic energy. For example, the metabolic energy of unconstrained reaching has been determined to be an increasing function of distance and a convex function of time [9], therefore predicting that the optimal movement duration increases with distance and with temporal discounting (Box 1). Although this may be consistent with experimental observations in some cases, optimizing energy alone cannot explain why other criteria exert a significant influence on movement. First, it is well known that large rewards yield faster movements [13]. Second, some movement parameters, such as the optimal duration for an isometric force contraction (Box 1), cannot be determined by optimizing energy alone, as energy in this case increases linearly with duration and intensity. Finally, the many formulations of the rate of consumption of metabolic energy for different kinds of movements and conditions strongly suggest that a criterion of movement optimality based on metabolic energy alone is unlikely.

**Box 1. Definitions.**

Utility: measure of desirability for 1 option/movement, quantified by the difference of benefits and costs.

Temporal discount: devaluation of benefits and costs due to the passage of time, e.g., US$10 is not worth the same if delivered immediately as after 1 year.

Effort discount: devaluation of the benefit associated with an option due to its associated effort expenditure.

Indifference point: Specific level of desirability at which 2 options are reported to be perceived as evenly desirable.

Isometric force contraction: contraction during which the muscles involved are held at a constant length.

**Fig 1:**
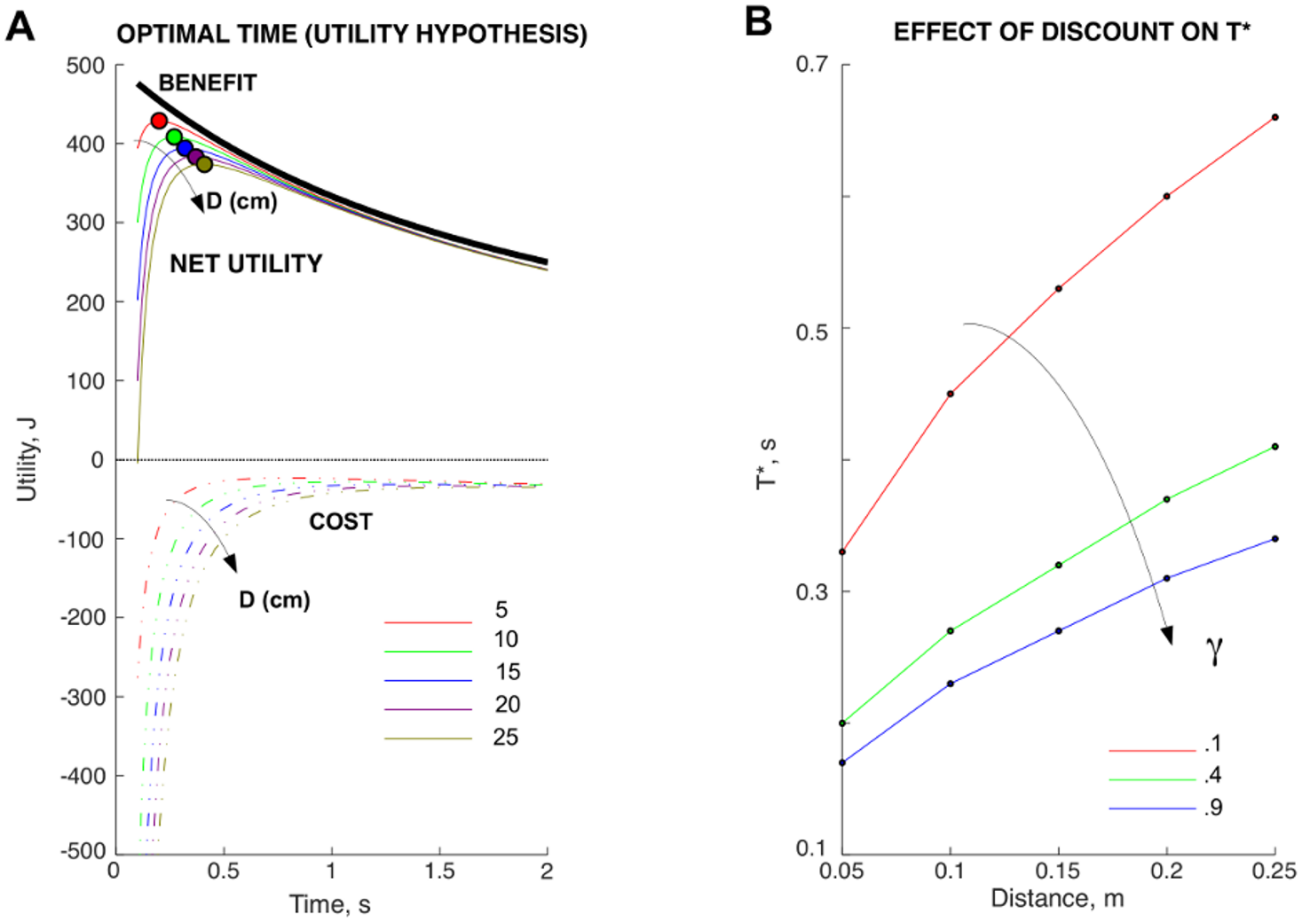
**(A)** Utility function traces (solid traces) resulting from trading off benefits (black solid trace) minus their associated costs (dashed-dot traces) as a function of movement time (T) for reaching movements of path distance (D) between 5 and 25 cm. The dots on the utility traces indicate the optimal time (T*) resulting from maximizing utility for that specific reaching, which increases with distance. **(B)** Effect of temporal discount (γ) on utility. Optimal movement times derived by maximizing utility are plotted as a function of distance for 3 specific temporal discount values. Movement times decrease as discount rates increase.

By contrast, reviewing movement in the context of embodiment necessarily introduces the notion of goal directedness [14], thereby leading to the second hypothesis of effort discounting. Importantly, decisions in this context are not driven by the subjectively perceived effort of each option alone but rather by their utility (Box 1), i.e., the difference between perceived benefits and costs. In other words, if movement is a necessary component of behavior, the specifics of movement should also abide by wagers of benefits and costs aimed at attaining a more favorable state. The supporting evidence is that decisions between movements hinge on the constituent parameters of movements themselves [11,15] and that generative models implementing this process, which quantify effort as a negative benefit, can be fit to a broad set of movement parameters and decisions between them [16,17]. Following this line of thought, Morel and colleagues show that models of decision-making assessing the logarithmic difference of the efforts associated with each movement option provide a significantly better fit of the subjects’ choices than those assessing the inverse of efforts, which is consistent with the notion of effort acting as a negative discount in the utility space. The negative discounting of effort reported by Morel and colleagues could therefore be viewed as the part of the subjective perception of effort that effectively influences motor control and decision-making. As an example, Fig 1A shows a set of utility curves plotted as functions of movement time, resulting from the optimization of the utility associated with unconstrained, free reaching movements [16]. In this case, trading benefits and costs yields an optimal movement time T*, which increases with distance and decreases with reward and discount (Fig 1B). Complementary to this, the results of Morel and colleagues go one step further in that their analysis extends to movements performed with a haptic manipulator that resists movement, consequently magnifying the influence of effort. Consistently with this, while the subjective effort of force production has been previously characterized as quasilinear in the case of isometric contraction [18], the results of Morel and colleagues provide compelling evidence that the relationship between force and effort in resistive reaching movements obeys a quadratic law. In summary, this suggests that for the same force exerted, resistive reaching is perceived as more effortful than isometric contraction and that the principles of effort perception are not well understood.

## Biomechanics, utility, and decision-making

**Fig 2:**
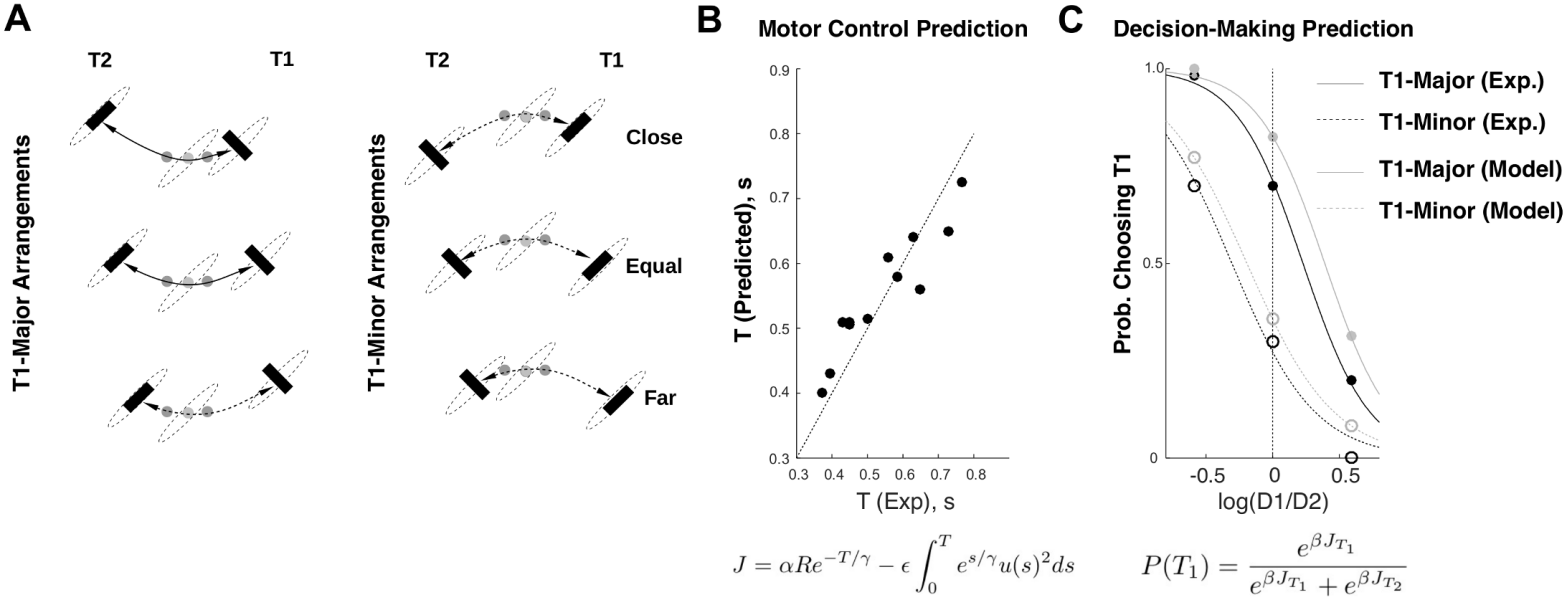
Effect of biomechanical costs in motor control and decision-making. **(A)** Decision-making task in which movements were aimed at the black rectangles. In each arrangement, the difference in biomechanical costs between T1 (right) and T2 (left) is maximal, although the relative path distance may vary. **(B)** Predicted versus measured average movement times for each of the 12 possible movements shown in **(A)**. The equation below is the utility function used to obtain the optimal trajectory (and movement time). **(C)** Predicted versus measured group patterns of choices for T1 as a function of relative target path distance (D1 and D2: path distances from the origin to T1 and T2, respectively) (see [11,15,19] for further detail). The predicted pattern is obtained by fitting the softmax temperature to the utilities (J) obtained for either movement and for each relative distance.

Biomechanical costs are movement expenditures caused by the structure of the motor apparatus and have been reported by Morel and colleagues insofar as outward movements were perceived as being more costly than inward ones. Although we cannot access the perception of these costs, their manifestation in the selection of motor parameters and between movements is also compliant with the same principle of utility optimization (see Fig 2).

Morel and colleagues determined that logarithmic differences of effort best fitted the subjects’ patterns of decision-making. Complementary to this, I next show an example of decisions between movements of different biomechanical cost being predicted by utility. To this end, I used a generative model and incorporated the cost of moving along each direction of movement as the subjective perception of effort (or effort discounting, Box 1). Utility is therefore a function of 3 parameters (Fig 2B, bottom): the scaling of reward (ρ), the sensitivity to effort (ε), and the temporal discount (γ). These were fitted to the times of experimental movements extracted from previous experiments in which subjects chose between movements with different biomechanical costs [19] (Fig 2A and 2B) (R^2^ = 0.90347; P = 5.62E−5). Next, the set of parameters obtained from fitting a metric purely of motor control (γ = 0.45, ε = 0.61, ρ = 25) was used to predict the pattern of experimentally observed movement preferences as a difference of the predicted utility of each movement (Fig 2C).

Although the analysis is based on group-averaged data, the predicted pattern of decision-making matches the experimentally observed choices, thereby reinforcing a unified view of the role of perceived effort in motor control and decision-making, which extends from biomechanical costs to more elaborate forms of effort.

## Conclusion

Morel and colleagues have performed experiments aimed at characterizing how physical effort is perceived subjectively [10] by asking subjects to decide between movements that differ incrementally in cost and quantifying the influence of the subjective perception of effort on these decisions. Their results show that despite the increase in mechanical work associated with movements with longer path lengths, the perceived effort associated with these movements does not vary if their duration remains constant. By contrast, movements of longer durations are perceived as more effortful regardless of path length. In other words, only time—and not distance—increases the perception of effort, strongly suggesting that effort is most likely integrated over time. Moreover, their results also suggest that the formulation relating moving force amplitude to perceived effort follows a quadratic law. This is the first specific formulation of moving force amplitude for transport movements, which diverges from the linear relationship observed for isometric force contractions [20].

In conclusion, the results of Morel and colleagues strongly argue against the idea that metabolic energy is the common determinant of both motor control and decisions between actions, as energy increases linearly with time and depends both on force and speed of contraction. By contrast, a more elaborate conceptualization of control costs centered around the notion of perceived effort seems increasingly likely. Thus, viewing effort perception from the perspective of effort discounting provides a unified framework from which to study the subjective perception of effort—one in which motor-control theory and decision-making can be directly related. The study of effort perception viewed as effort discounting has proceeded incrementally from metabolic energy to effort discounting and utility, thereby establishing a principled backbone for relating motor-control theory to decision-making. However, further research is required to find a general formulation of effort perception that encompasses many different tasks and conditions. Consequently, a future challenge will be to find independent measures of effort that can dissociate the subjective perception of effort and its sizeable influences on behavior.
