# Correction: Perceived Effort for Motor Control and Decision-Making

**DOI:** 10.1371/journal.pbio.1002617

**Published:** 2017-11-22

**Authors:** Ignasi Cos

In the Funding section, the grant number from the funder Marie Sklodowska-Curie Research Grant Scheme is listed incorrectly. The correct grant number is: IF-656262.
